# Changes in Disability Levels Among Older Adults Experiencing Adverse Events in Postacute Rehabilitation Care: A Prospective Observational Study

**DOI:** 10.1097/MD.0000000000000570

**Published:** 2015-02-27

**Authors:** Mariano Gacto-Sánchez, Francesc Medina-Mirapeix, Esther Navarro-Pujalte, Pilar Escolar-Reina

**Affiliations:** From the Department of Physical Therapy (MGS), EUSES University School, University of Girona, Girona, Spain; Department of Physical Therapy (FMM, PER), University of Murcia, Murcia, Spain; and Department of Education (ENP), Region of Murcia, Murcia, Spain.

## Abstract

This study aimed to assess the relationship between adverse events (AEs) and changes in the levels of disability from admission to discharge during inpatient rehabilitation programs.

A prospective cohort study was conducted among a cohort of inpatients (216 older adults) admitted to a rehabilitation unit. The occurrences of any AE were reported. The level of disability regarding mobility activities was estimated using the disability qualifiers from the International Classification of Functioning, Disability, and Health. Changes in the levels of disability between admission and discharge were assessed. Baseline-measured covariates were also selected.

Regarding all 4 disability levels (“no limitation,” “mild,” “moderate,” “severe,” and “complete disability”), a total of 159 participants experienced an improvement at discharge (126 participants progressed 1 level, whereas 33 improved 2 disability levels), 56 made no change, and no participants experienced a decline.

The occurrence of fall-related events and the diagnostic group (musculoskeletal system) are specific predictive factors of change in the level of disability. The odds of undergoing a change in any disability level between admission and discharge decreases by 68% (1–0.32) when patients experience fall-related events (odds ratio [OR] = 0.32, 95% confidence interval [CI] = 0.11–0.97, *P* = 0.041) and increases for individuals with musculoskeletal conditions (OR = 3.91, 95% CI = 1.34–11.38, *P* = 0.012).

Our findings suggest that increased efforts to prevent the occurrence of these AEs, together with early interventions suited to the diagnosis of the affected system, may have a positive influence on the improvement of disability. Further studies should evaluate disability over time after discharge to obtain a better sense of how transient or permanent the associated disability may be.

## INTRODUCTION

Most hospitalized older adults suffering any acute injury or disease are vulnerable to transient or permanent disability after acute hospital care.^1^ In this respect, there is a clear trend toward providing rehabilitation care in dedicated units of an acute care hospital or specialized rehabilitation facilities in order to optimize functioning after acute care.^2–4^ At the same time, there is a tendency toward identifying risk factors of rehabilitation failure, such as poor functional status at discharge.^5^

Adverse events (AEs) are health care-associated incidents that cause harm.^6^ Several studies have previously shown that a substantial number of older adults in acute hospital units (eg, surgery, intensive care, and others) experience AEs.^7–9^ Most of these AEs contribute to negative consequences regarding mortality, length of hospital stay, or functional status at discharge.^10,11^ Based on these findings, there is growing interest in improving patient safety and reducing the number of AEs in acute hospitals.^12,13^ However, the relationship between AEs and functional status has not been explored in older individuals receiving postacute rehabilitation care in acute hospitals. Although existing literature provides some insight into what type of consequences are likely to be experienced by patients with AEs in acute hospitals, their frequency or degree of severity in rehabilitation units could widely differ.

This study assessed the relationship between AEs and changes affecting the level of disability between admission and discharge during inpatient rehabilitation programs in a cohort of older adults who were admitted to a rehabilitation unit of a French acute hospital. A group was studied that composed of hospitalized older adults who experienced any AE during their stay at the rehabilitation unit. We hypothesized that at discharge from the rehabilitation unit, such group would be less likely to improve their initial level of disability.

## METHODS

### Study Design and Setting

We conducted a prospective cohort study at a public hospital in Castelnaudary (France). This hospital, with its 268-bed capacity, provides acute and postacute care. It has a rehabilitation unit for early postacute rehabilitation of patients transferred either from its acute services or other acute hospitals. All their inpatients are included in a postacute rehabilitation program, which usually requires a standard stay of 4 weeks: the hospital established this standard period based on the average usual lengths of stay and economic criteria. An interdisciplinary team of physicians, nurses, and therapists, specialized in rehabilitation care, cooperate in managing the demands of early postacute rehabilitation and the criteria for discharge, based on each patient's physiological and functional stability, and also considering the sociofamiliar context. The institutional review board of the hospital approved this study protocol.

### Study Population and Recruitment

Inpatients over 65 years of age were recruited to the study provided that they had been admitted to the rehabilitation unit and were included in a postacute rehabilitation program. Individuals were excluded if they had uncorrected visual impairment or inability to understand simple instructions required for filling questionnaires.

During an 11-month period (between September 2011 and July 2012), a consecutive sample of the entire accessible population was recruited within the first 24 hours of their inclusion into the rehabilitation program by a provider who assessed their eligibility criteria. The number and reasons for exclusion were documented. An informed consent form was obtained from all participants. Demographic data (age and sex) and reasons for hospital admission (diagnostic) were provided by means of the medical records. Participants were classified into 3 major diagnostic groups: musculoskeletal conditions (eg, joint replacements, fractures, and others); cardiopulmonary conditions (eg, pneumonia, pulmonary edema, and others); and medically complex conditions (eg, debility resulting from illness, stroke or other complex neurologic conditions, and others).

### Measurements

#### Adverse Events

For this study, the hospital used the indicators for patient safety in the Organisation for Economic Co-operation and Development (OECD) countries as a framework for reporting AEs.^14^ This framework included 4 types of relevant AEs: infections (ventilator pneumonia, wound infections, infections due to medical care, decubitus ulcers); postoperative complications (complications of anesthesia, postoperative hip fracture, postoperative pulmonary embolism or deep venous thrombosis, postoperative sepsis, technical difficulty with procedure); sentinel events (transfusion reaction, wrong blood type, wrong site surgery, foreign body left-in during procedure, medical equipment-related AEs, medication errors); and other care-related AEs (patient falls, in-hospital hip fracture or fall), henceforth mentioned as “fall-related events” or “fall events.”

Members from the multidisciplinary health team were trained to report the occurrence of any AE during hospital stay at the rehabilitation unit. Thus, AEs were reported and briefly described by the observer. In weekly meetings, medical record of each patient was reviewed by 2 clinical researchers in order to evaluate the AEs that occurred during hospital stay. When the 2 researchers disagreed on the presence of AEs, they interviewed the clinical staff and started a process to achieve consensus.

#### Outcome Measures

The change in disability levels between admission and discharge was the outcome measure. To estimate the level of disability both at admission and at discharge, we used the “mobility activities” domain from the International Classification of Functioning, Disability, and Health (ICF) as a framework^15^ together with the ICF qualifiers (“no limitation,” “mild,” “moderate,” “severe,” and “complete disability”). The extent of disability for mobility activities was initially measured with the Mobam-in instrument^16^ and afterward patients’ scores were used to estimate the ICF disability qualifier according to a previously validated procedure and using the ICF category interval scale (0–100)^17^ within the framework of the ICF classification for describing functioning and disability.^18^

The Mobam-in is an instrument developed to be used only with inpatients and consists of activities typically performed in this environment. The Mobam-in covers 2 domains of functioning in mobility activities: upper and lower body mobility.^16,19^ The lower body domain was selected as the main outcome measure of this study. The upper body domain was alternatively used only when participants exclusively had musculoskeletal impairments affecting the upper extremities. The Mobam-in scores are based on a 0-to-100 interval scale, where lower scores imply more limitation. Individuals obtaining a score between 0 and 4 were assigned to the qualifier “complete disability”; those with a score between 5 and 49.9 were qualified as “severe”; participants with a score between 50 and 74.9 were allocated to the qualifier “moderate”; those with a score between 75 and 94.9 were “mild”; and those with a score between 95 and 100 were allocated to the qualifier “no disability.”

#### Demographic and Health-Related Variables

A total of 7 variables were selected from the literature research as covariates, based on their potential association, either with the occurrence of AEs in hospitalized seniors or with disability at discharge. These variables were measured at baseline and classified into 3 domains: demographic, clinical, and functional factors. The demographic domain included age (years) and gender. The clinical domain included the diagnostic group, the number of medications or prescription drugs used at the beginning of rehabilitation program, and the number of comorbidities, measured using the Functional Comorbidity Index.^20^ The functional factors domain included frailty and fear of falling. Frailty was measured by means of the Reported Edmonton Frail Scale, based on a scale from 0 to 18, where higher scores entail more severe frailty.^21^ The extent of fear of falling was measured using the Short Falls Efficacy Scale International, which ranges from 7 to 28, where higher scores reflect a more severe concern of falling.^22^ Moreover, as the outcome of change in the level of disability depends on Mobam-in scores, we also added the Mobam-in score at baseline as a possible factor, because of the fact that it could be a potential confounder.

### Statistical Analyses

Descriptive statistics were used to characterize the cohort at baseline. We used Pearson χ^2^ and independent *t*-test to examine differences in baseline characteristics with respect to the occurrence or absence of AEs.

Change was assessed by comparing the levels of disability level for each person. The results were categorized into 3 patterns: improvement, no change, and decline. In addition, participants with improvement patterns were classified based on the number of improved disability levels (eg, 1 level when participants changed from the “severe” to the “moderate” disability level). Participants whose individual Mobam-in scores changed by less than the minimal detectable change (MDC) were all included in the no-change category. The MDC, also known as the reliable change index,^23^ is considered the minimal amount that is not likely to be due to variability accounted by measurement error. We calculated the MDC as described elsewhere^24^ and using the test–retest reliable coefficient reported in a previous study.^16^ In addition, participants with improvement patterns were classified based on the number of improved disability levels (eg, 1 level when participants changed from the “severe” to the “moderate” disability level).

Univariate and multivariate logistic regression analyses were used to assess the possible factors associated with the change in disability levels. In the univariate analyses, associations were tested for a significant relationship (*P* < 0.05) with the change in disability levels. Because of the small incidence rates of AEs, these were grouped as follows: “fall events” and “other” (infections and/or sentinel events, as we considered that both groups shared the same need for additional interventions following the AE). In the multivariate analysis, the factors with statistically significant contributions concerning both the exposure (AEs) and the outcome measure (changes in disability levels) were combined and used as independent variables, whereas “change” (improvement = 1, no change = 0) was the dependent variable. The final model was produced by the enter method. Goodness-of-fit and regression diagnostics for the model were assessed using methods described elsewhere.^25^ Analyses were performed using the statistical package SPSS version 19.0, IBM Corp., Armonk, NY.

## RESULTS

A total of 230 participants were identified during the study period. Of these, 12 were excluded (3 of these had uncorrected visual impairment, whereas 9 were unable to understand simple instructions). Two patients refused to participate. Thus, 216 participants were considered, of which 65.7% (142 participants) were admitted from acute units of other referral hospitals.

Participants’ characteristics at baseline are described in Table 1. Musculoskeletal system disorders were present in 66.2% of participants (40 participants had impairment exclusively in the upper extremity). A total of 49.5% of the sample showed 1 or 2 comorbidities, whereas 3.2% had ≥3 health conditions. The most common comorbidities were hypertension (20.1%), diabetes (18.7%), and osteoporosis (16.7%). Disability measures at baseline disclosed that 93.9% of participants admitted to the rehabilitation program showed moderate-to-severe levels of disability. Participants who experienced AEs were older, more likely to be diagnosed as medically complex and showed higher levels of frailty and a greater number of comorbidities.

**TABLE 1:**
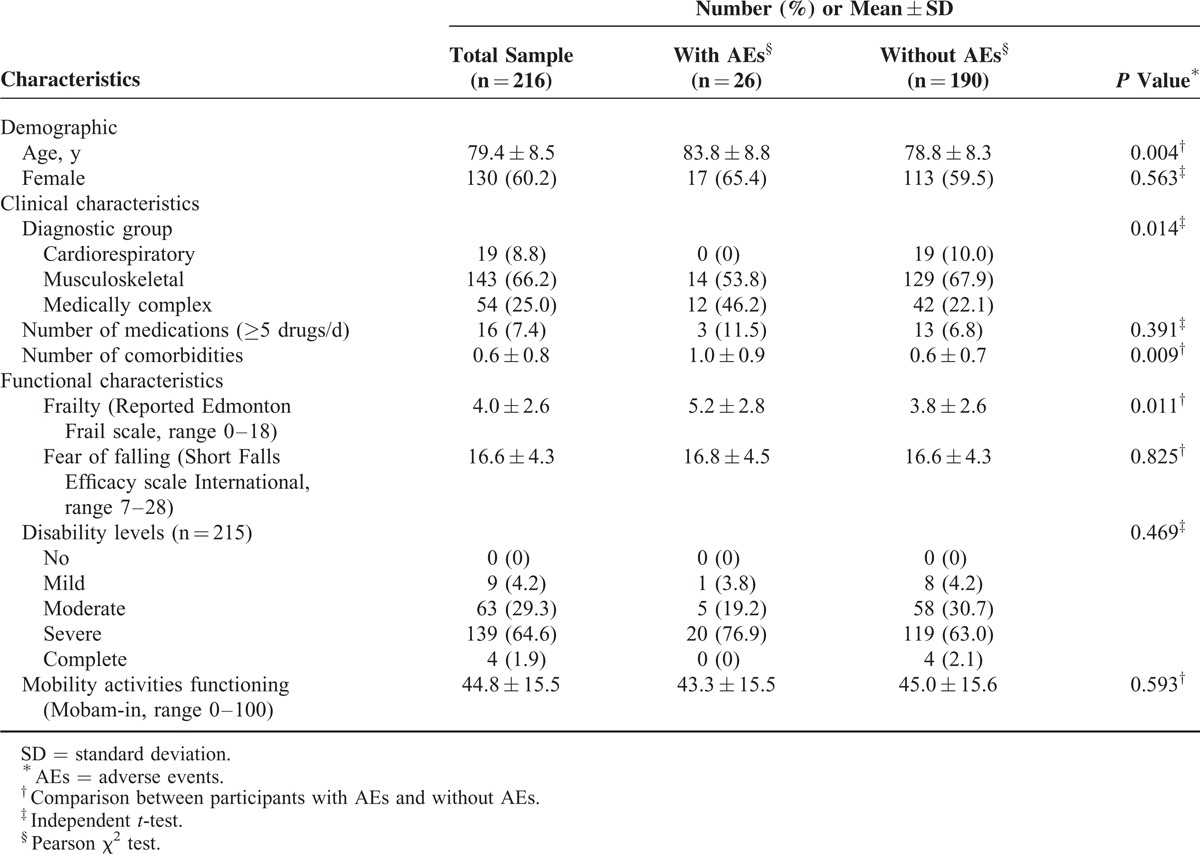
Participant Characteristics at Admission (n = 216)

A total of 26 participants (12.0%) experienced at least 1 AE during their hospital stay. Most of them (25) experienced just 1 AE. Experiences of AEs were more frequent in fall-related events (8.3%), while infections (1.9%) and sentinel events (1.9%) were reported less frequently. No postoperative complications were identified.

**FIGURE 1:**
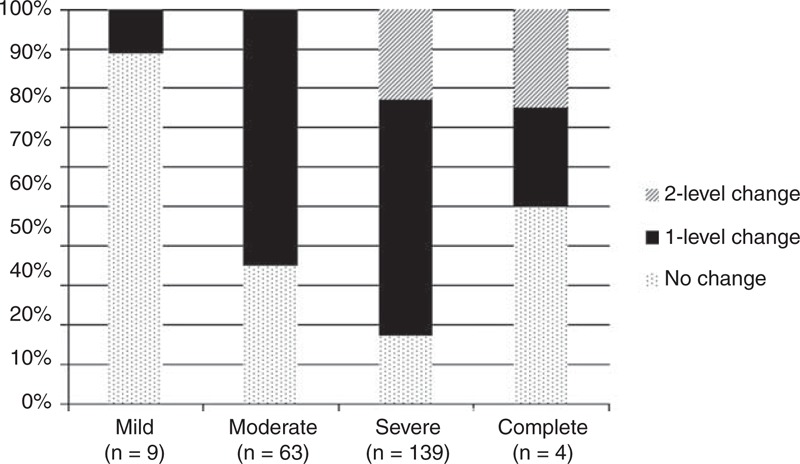
Proportion of the sample exhibiting patterns of change for the specified disability levels at admission.

Figure 1 displays the change in disability levels for each level at admission. It illustrates the proportion of participants whose level did not change, those who improved 1 disability level (ie, from “severe” to “moderate,” for instance), and those who improved 2 levels (ie, from “severe” to “mild,” for example). Participants with mild and complete disability displayed a lower percentage of change. In contrast, participants with severe and moderate disability demonstrated a greater level of change, with a higher proportion of participants improving 1 level. Regarding all 4 disability levels, a total of 159 participants experienced an improvement pattern (126 participants progressed 1 level, while 33 improved 2 disability levels), 56 made no change, and no participants demonstrated a decline. Consequently, at discharge, 2 (0.9%) of the participants had complete disability, while severe disability was present in 25 participants (11.6%). A total of 106 participants (49.3%) had a moderate disability level, whereas 81 participants (37.7%) reported mild levels. One (0.4%) of the participants reported no disability.

Results of the univariate analyses are presented in Table 2. These unadjusted analyses provide a relative measure of the odds of experiencing a change in the level of disability between participants with specific characteristics compared with those without these characteristics. For example, the musculoskeletal subgroup was significant when using the cardiorespiratory subgroup as the reference in the analysis. Based on both these results and the significant relations (*P* < 0.05) shown in Table 1, 5 factors entered into the multivariate model: age, diagnostic group, comorbidities, frailty, and occurrence of AEs.

**TABLE 2:**
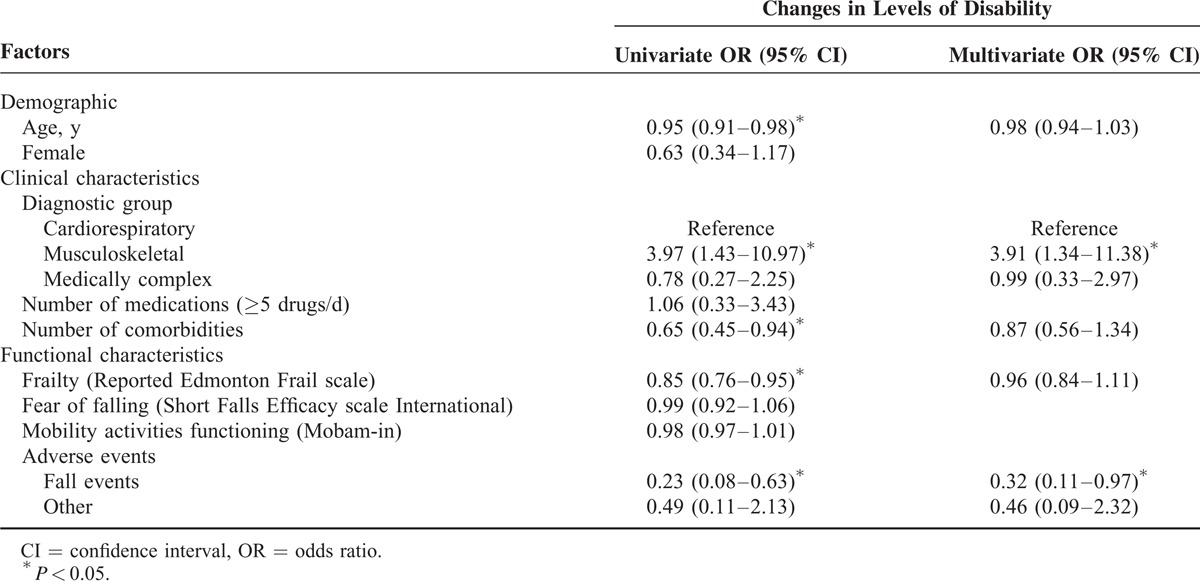
Summary of Univariate and Multivariate Logistic Regression Analyses With Changes in Levels of Disability as the Dependent Variable

Table 2 also presents the results from the multivariate analysis. According to the model, the odds of undergoing a change in the level of disability between admission and discharge decreases 68% (1–0.32) when patients experience fall-related events (odds ratio [OR] = 0.32, 95% confidence interval [CI] = 0.11–0.97, *P* = 0.041) and increases for individuals with musculoskeletal conditions (OR = 3.91, 95% CI = 1.34–11.38, *P* = 0.012).

## DISCUSSION

Fall-related AEs occurring during hospital stay at a rehabilitation unit were important independent risk factors for changes in disability levels between admission and discharge in a cohort of older adults. In this study, participants who suffered fall-related AEs during their stay at the rehabilitation unit experienced around 3 times lower odds of improving their levels of reported disability between admission and discharge. In contrast, patients admitted with a musculoskeletal condition diagnosis had around 4 times higher odds of improving their level of disability.

To our knowledge, this is the first study to explore the association between AEs and their consequences on changes in disability levels. Findings from our study have implications for the improvement of the safety of hospitalized older adults and the subsequent consequences for rehabilitation units of acute care hospitals. Overall, efforts should be channeled into improving fall-prevention strategies^26–29^ as well as in developing early interventions for any potential AE with the aim of identifying individuals at a higher risk of not improving their disability level between admission and discharge. Concerning these participants, interventions could potentially lower the risk of subsequent high disability and even the economic costs linked to longer hospital stays.^30^

Our findings show that AEs were experienced by nearly 12% of the older patients in the sample. These rates are consistent with other studies of elderly patients hospitalized in nonsurgical departments.^8,12^ However, in patients participating in a rehabilitation program, AEs have not been previously tested. These patients usually show high levels of disability upon admission to rehabilitation units, and their eligibility is often based on the fact that their functional status may likely be optimized during their stay. The fact that these patients experience AEs at all seems contradictory to the goal of rehabilitation units, through which disability decreases.^31^

A high percentage of participants improved their initial disability level. Nevertheless, the occurrence of moderate–severe disability among participants at discharge was higher than previously reported.^8,12^ This observation might be based on differences in the accuracy of the measurement of disability. Although previous studies are often retrospective and highly dependent on medical records and the reviewer's judgments,^12^ we measured disability by means of patient self-reported questionnaires. Finally, an alternative and likely reason would be that patients participating in rehabilitation units usually have poorer functional status at discharge than individuals in other acute departments.

This study shows that there is room for improvement in the adoption of fall prevention measures. Our study also highlights the importance of the diagnostic group as a means of identifying patients at a higher risk of poor recovery based on the reported levels of disability at admission. Tailored interventions within the framework of rehabilitation should therefore be introduced into clinical practice based on these diagnostic groups, in order to enhance their functional status and provide an improved recovery progression from admission to discharge. Nevertheless, in order to formulate more specific interventions, further research is warranted for a better understanding of other modifiable factors that may explain the incidence of AEs.

### Strengths and Limitations of the Study

A strong aspect of the study is the prospective design and the use of an approach that combines medical record reviews with methods in which clinical staff and participants are interviewed. Prospective studies show advantages over retrospective studies for estimating AEs and their consequences, as they can determine more events and are more reliable.^32^ Moreover, record reviews may not be the most accurate method to obtain insight into AEs, since they are less often reported in patient records. Hence, the interview with clinical staff may compensate for information bias in patient records.^33,34^

Our findings should be interpreted in light of our study's methodological limitations. First, the types of AEs studied were those proposed by the framework of the OECD, which was designed for acute hospitals. Although our study took place in an acute hospital, it is possible that our results represent an overly optimistic view of the extent of AEs in rehabilitation units if additional and more specific AEs occurred. Second, our sample was recruited from patients in a rehabilitation unit of a large public French hospital, who may differ from other older individuals receiving rehabilitation in other postacute care settings or other health care systems. Therefore, until further research is conducted on a broader sample, these results should be generalized cautiously.

## CONCLUSIONS

In summary, this study found that older adults who experienced an AE during rehabilitation displayed more frequently poorer improvement rates concerning levels of disability from admission to discharge. Our results suggest that efforts to prevent the occurrence of these AEs, as well as early interventions based on the diagnosis of the affected system, may have a positive influence on the improvement of disability levels. Further studies should evaluate disability over time after discharge to obtain a better sense of how transient or permanent the associated disability may be.
